# Beyond Pathogens: Modulating the Commensal Microbiota to Reshape Immunity for Respiratory Disease Intervention

**DOI:** 10.3390/pathogens15090883

**Published:** 2026-08-23

**Authors:** Yang Yang, Jinglei Cui, Xinyue Dong, Yunhan Huang, Jiayao An, Tianyi Cui, Xiangqin Ou, Tao Liu, Xiumei Gao, Han Zhang, Xin Zhao

**Affiliations:** 1 Ministry of Education Key Laboratory of Pharmacology of Traditional Chinese Medical Formulae, Tianjin University of Traditional Chinese Medicine, 10 Poyanghu Road, Tianjin 301617, China; 2 State Key Laboratory of Chinese Medicine Modernization, Tianjin University of Traditional Chinese Medicine, 10 Poyanghu Road, Tianjin 301617, China; 3 School of Medical Technology, Tianjin University of Traditional Chinese Medicine, 10 Poyanghu Road, Tianjin 301617, China

**Keywords:** respiratory tract microbiota, immunity, respiratory disease, disease management

## Abstract

The respiratory tract microbiota has been recognized as a niche with complex and diverse microbial communities, the composition and function of which in the state of health and disease have become increasingly clear. Relevant literature on respiratory tract microbiota and immune and respiratory disease published in PubMed, SpringerLink and Web of Science up to June 2026 was narratively reviewed. This review aims to elucidate the interactions between the commensal microbiota of the respiratory tract and the host immune system and reveal how these interactions evolve from homeostasis to disease pathogenesis. We also analyze how dysbiosis can promote chronic airway inflammation by changing microbial metabolites, destroying the integrity of epithelial barrier, and activating abnormal immune and inflammatory pathways, thus playing a key driving role in chronic obstructive pulmonary disease (COPD), asthma, cystic fibrosis (CF), bronchiectasis and other diseases. Additionally, the potential and challenges of current treatment strategies based on microbiota regulation, such as herbal medicines and probiotics, in the prevention and treatment of respiratory diseases are discussed. This review provides a new perspective for revealing the etiology of respiratory diseases and lays a solid theoretical foundation for the development of microbial intervention therapy.

## 1. Introduction

**Figure 1:**
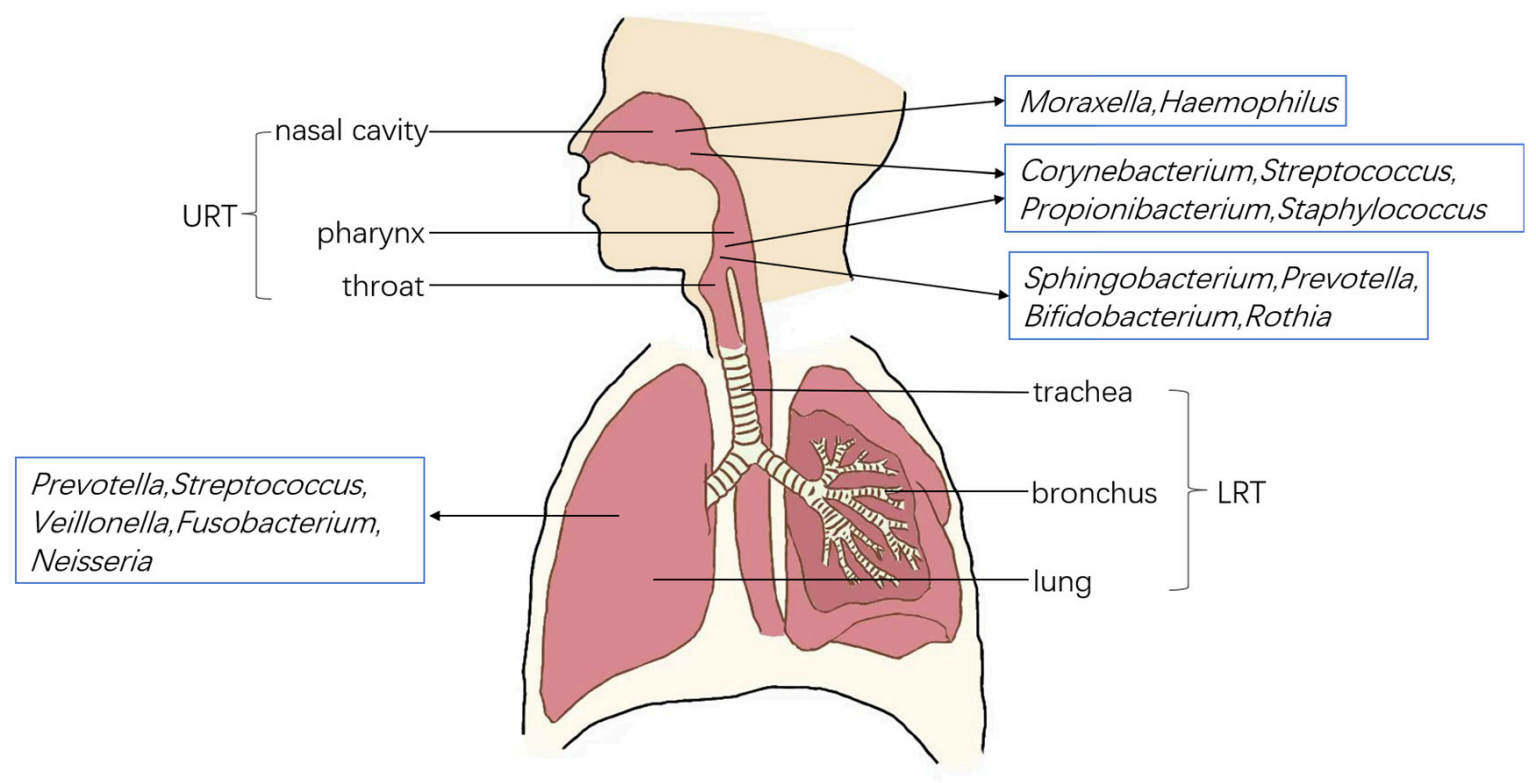
Distribution of microbiota in the respiratory tract. (URT: upper respiratory tract; LRT: lower respiratory tract).

The respiratory tract microbiota not only participates in the development and maturation of the mucosal barrier but also plays a key role in resisting pathogen invasion, inhibiting excessive inflammation and preventing allergic diseases by regulating the host’s innate and adaptive immune responses [1]. The upper respiratory tract (such as nasal cavity, pharynx) and lower respiratory tract (such as trachea, bronchus and lungs) together constitute a heterogeneous niche for respiratory tract microbiota colonization, and their composition and density are significantly different (Figure 1), which are influenced by factors such as temperature, humidity, pH, partial pressure of oxygen, and nutrient availability. Higher partial pressure of oxygen and reactive oxygen species inhibit anaerobic bacteria and sensitive aerobic bacteria; dynamic changes in pH modulate the properties of mucus and the activity of antimicrobial peptides; and mucus-rich areas and specific glycosylated receptors promote bacterial adhesion and biofilm formation. At the same time, airway secretions provide carbon sources and attachment sites that support microbial survival, while antimicrobial peptides (such as surfactins, defensins, and lactoferrin) exert selective pressure on the composition of the microbial community. These physicochemical and biochemical determinants collectively shape the diversity, distribution, and stability of the respiratory tract microbiota [2]. The most abundant genera were *Prevotella*, *Streptococcus*, *Veillonella*, *Neisseria*, *Haemophilus* and *Fusobacterium* [3], which have been clinically reported to be related to bronchiectasis [4], pneumonia [5], and COPD [6]. Among COPD patients hospitalized for acute exacerbations, the presence of *Staphylococcus* in sputum was significantly associated with the risk of death [7]. *Staphylococcus* enrichment signifies pulmonary dysbiosis and immune imbalance, and patients also exhibit a loss of microbial diversity and a reduction in commensal bacteria such as *Veillonella* [7]. The dysbiosis is associated with the disruption in the host’s immune defenses, thereby creating conditions conducive to the overgrowth of opportunistic pathogens. Therefore, it is of great scientific value and clinical significance to analyze the interaction network between respiratory tract microbiota and host immunity for developing diagnostic and therapeutic strategies.

Compared with transiently passing bacteria, the stable resident microbiota forms a closer interaction relationship through long-term coevolution with the host. They train and interact with the host immune system by continuously releasing microbial-associated molecular patterns (MAMPs), such as lipopolysaccharide (LPS) and peptidoglycan [8]. These signals can guide the functional polarization of alveolar macrophages to maintain their tolerance in the resting state, and at the same time, when they are encountering pathogens, they can quickly start an effective antibacterial response; they also regulate dendritic cell (DC) maturation and antigen presentation to T cells, thereby directing Th1, Th2, or Treg differentiation and establishing a precise immune balance [9]. However, different bacterial species—and even different strains of the same species—exert significantly different effects on host gene expression, particularly the interferon response [10], and the spatial distribution of interactions between the microbiota and the immune system is also highly refined. For example, in patients with COPD, various pathogens such as *Haemophilus* and *Streptococcus* are involved in exacerbating symptoms [11]. Additionally, bacteria may interact with different types of immune cells. During infection with *Mycobacterium tuberculosis* (*Mtb*), it can both stimulate DCs to secrete cytokines and regulate T-cell responses [12,13,14]. A previous review summarized respiratory fungal microbiota–immune interactions, showing that respiratory fungi can directly manipulate host immunity and influence the severity and frequency of chronic airway disease exacerbations [15]. Pichon et al. [16] reviewed the impact of the respiratory tract microbiota on host responses to respiratory viral infection, including regulating viral release/adherence and immune responses. Baker et al. [17] elucidated how the lung microbiota precisely shapes immune homeostasis. However, the network between the microbiota and immune system in the upper and lower respiratory tract remains unclear, and therapeutic targets based on this network have yet to be identified.

Based on existing evidence, this review aims to elucidate the network of interactions between the respiratory tract microbiota and five major immune cell types, including DCs, macrophages, neutrophils, T cells, and B cells. We further discuss the distinct microbiota–immune interaction patterns across various respiratory diseases, like COPD, asthma, CF, and tuberculosis (TB), using the integrated network framework, and provide potential guidance for future research directions and therapeutic strategies.

## 2. The Regulatory Mechanism of Respiratory Tract Microbiota on Immune Cells

The upper respiratory tract microbiota is mainly composed of *Staphylococcus*, *Propionibacterium*, *Dolosigranulum*, *Corynebacterium*, *Moraxella*, *Streptococcus*, *Haemophilus*, *Rothia*, *Veillonella*, and *Prevotella* [18,19,20]. The most common bacterial genera in the nasal cavity are *Corynebacterium*, *Propionibacterium*, *Streptococcus*, *Staphylococcus*, *Moraxella*, and *Haemophilus* [21,22]. The common bacterial genera in the throat are mainly *Staphylococcus*, *Streptococcus*, *Sphingobacterium*, *Prevotella*, *Corynebacterium*, *Bifidobacterium*, *Rothia*, and *Propionibacterium* [23]. The composition of the lower respiratory tract microbiota is basically the same as that of the upper respiratory tract. At the phylum level, it is mainly composed of *Firmicutes*, *Bacteroidetes*, *Proteobacteria*, and *Actinobacteria* [24]. At the genus level, it is mainly composed of *Prevotella*, *Streptococcus*, *Veillonella*, *Clostridium*, and *Neisseria* genera [25,26]. Some bacteria, such as *Haemophilus*, *Prevotella*, and *Veillonella* [27], can interact with immune cells through pattern recognition receptors (such as Toll-like receptors, TLRs); other bacteria, such as *Staphylococcus* [28], can directly or indirectly affect the differentiation and function of T cells. The microbiota mentioned above represents some of the key components in the host–microbiota immune interaction network.

### 2.1. Innate Immune System

When the physical barrier of the respiratory tract (such as tight junctions, mucus layer or cilia clearance function) is damaged, this may not only increase the risk of pathogen colonization but also cause the disorder of symbiotic microbiota and destroy immune homeostasis. This process activates bone marrow-derived immune cells (such as macrophages, neutrophils) and DCs through pattern recognition receptors (PRRs, such as TLRs and NOD-like receptors), triggers NF-κB and interferon signaling pathways, promotes the release of pro-inflammatory factors (such as IL-1β, TNF-α and IL-6), and thus initiates an acute inflammatory response. The composition of the microbiota and the functional state of the innate immune system collectively maintain immune homeostasis in the lungs, thereby regulating the intensity of inflammatory responses.

#### 2.1.1. Dendritic Cells (DCs)

DCs are antigen-presenting cells, which can play a central role in bridging innate and adaptive immunity by guiding the development of antigen-specific T-cell responses. In vitro co-culture study has shown that [29] *Haemophilus influenzae B*, non-typeable *Haemophilus influenzae* (NTHi), *Moraxella catarrhalis*, *Prevotella melaninogenica*, *Hoylesella nanceiensis*, *Segatella salivae*, *Veillonella dispar*, *Actinomyces graevenitzii* and *Actinomyces oris* can induce DCs to mature, thus secreting cytokines and inducing inflammation. The levels of IL-23, IL-12p70 and IL-10 induced by pathogenic *Proteus* were higher than those of other symbiotic bacteria. The levels of IL-23 and IL-12p70 induced by *Actinomyces* were the lowest, and the levels of cytokines induced by *Actinomyces* were like those of immature DCs. *Prevotella* could partly reduce the production of IL-12p70 in DCs induced by *Haemophilus*. Animal models have demonstrated that *Mtb* produces a functional protein called alpha-crystallin (Acr); however, it is worth noting that this protein plays a dual role at different stages of DC development (pre-mature and post-mature): when immature DCs are exposed to Acr during early differentiation, intracellular SOCS-3 expression increases significantly. High levels of SOCS-3 strongly inhibit STAT-1 phosphorylation. STAT-1 is a key transcription factor that promotes DC maturation and the secretion of pro-inflammatory cytokines (such as IL-12); when it is inhibited, DCs cannot mature normally and exhibit an immunosuppressive state. When mature DCs are exposed to Acr1, the opposite occurs. At this point, SOCS-3 levels are very low, and STAT-1 is strongly activated and phosphorylated. Consequently, DCs can further mature and efficiently secrete cytokines and upregulate CCR7 [12]. *Mtb* and *Yersinia pestis* could regulate the chemokine response of DCs by an IL-12p40-dependent mechanism, indicating the extensive influence of this mechanism in the induction of the cell response [30]. In patients with CF, the expression of C-type lectin CD301 is low in myeloid dendritic cells (mDCs), and the level of CD301 on mDCs may also be positively correlated with the relative abundance of *Mycobacterium* and *Staphylococcus* [31]. Thus, the intervention of microbiota–DCs crosstalk may contribute to the transition from acute inflammation to chronic disease.

#### 2.1.2. Macrophage

Macrophages are derived from hematopoietic stem cells in bone marrow. They initially differentiate into monocytes, enter the bloodstream, then migrate to tissues and differentiate into macrophages, which are involved in the mononuclear phagocyte system and possess powerful phagocytic, antigen presentation and immune regulation functions. Phagocytosis is triggered by the interaction between PRRs expressed on the surface of macrophages and pathogen-associated molecular patterns (PAMPs) present on the surface of pathogens. TLRs are a type of PRR that participate in the recognition of bacteria and in the subsequent signaling responses within macrophages (such as actin reorganization) [32]. This allows pathogens to be absorbed by phagocytes and promote the release of pro-inflammatory cytokines and chemokines, which recruit other immune cells such as neutrophils and eosinophils into the airways.

Macrophages can be polarized into classical activated (M1) and alternative activated (M2) macrophages. Dynamic changes in the M1/M2 polarization of macrophages largely shape the inflammatory microenvironment in the lungs and are closely associated with the processes of tissue damage and repair [33]. During severe infection or inflammation, macrophages initially adopt the M1 phenotype and, in response to stimuli, release pro-inflammatory cytokines, including TNF-α, IL-1β, IL-12, and IL-23 [34]. In vitro studies have shown that some commensal bacteria found in the airway, such as *Megasphaera*, *Streptococcus mutans* and *Streptococcus sanguis*, that produce short-chain fatty acids (SCFAs) could inhibit cytokine production and inflammation after LPS stimulates macrophages [35,36]. *Staphylococcus aureus* mediates the recruitment of CCR2^+^CD11b^+^blood monocytes into the alveoli and then differentiates into macrophages with anti-inflammatory phenotypes [37] in the mouse model. Different clinical isolated NTHi strains changed the monocyte-derived macrophage (MDM) response; compared with *S. aureus* strain *ST201*, *ST14* induced an increase in IL-10 and NF-κB expression [38] in vitro. NOD2 contributes to a protective inflammatory response in the lungs during *S. aureus*-induced pneumonia, as NOD2-knockout mice exhibit impaired macrophage and neutrophil recruitment, higher bacterial loads, and increased mortality [39]. *Bordetella pertussis* activates NLRP3 inflammasomes in human macrophages in vitro, resulting in caspase-mediated release of IL-18 and IL-1β [40]. Itaconate, an endogenous metabolite in the lungs, exerts anti-inflammatory and antioxidant effects through alveolar macrophages, inhibiting the activation of the mitochondrial NLRP3 inflammasome and oxidative stress [41] in an ovalbumin-induced mouse model. In a vancomycin-induced lung dysbiosis mouse model, SCFAs derived from the pulmonary microbiota, via the SCFA receptor FFAR2, are highly expressed on alveolar macrophages—they promote IL-1β production, inflammasome activation, and NF-κB phosphorylation in alveolar macrophages, thereby counteracting *Klebsiella pneumoniae* invasion [42]. The macrophage–microbiota crosstalk has been implicated in various infectious inflammatory conditions, where aberrant macrophage polarization and microbial dysbiosis mutually reinforce each other to enhance inflammation and impair pathogen clearance.

#### 2.1.3. Neutrophils

Neutrophils are the most abundant granulocytes in the innate immune system, accounting for 50–70% of circulating leukocytes. As the first line of defense against bacterial and fungal infections, they are differentiated from bone marrow hematopoietic stem cells and released into the blood after maturation. In the mouse model infected with methicillin-resistant *S. aureus*, the increase in circulating neutrophils is not limited to mature neutrophils but depends on the CD35^+^/CD49d^+^ double-positive subgroup, indicating that the relative balance of CD49d^+^ neutrophils is very important for controlling bacterial infection [43]. Neutrophils capture pathogens and control overwhelming infection by releasing neutrophil extracellular traps (Net), but the formation of net can lead to the death of respiratory epithelium and endothelial cells and accelerate the progress of COPD [44]. Clarke et al. showed that [45] NOD1 ligands derived from the gut microbiota can be transported into the bloodstream and bone marrow, where they enhance the protective function of peripheral neutrophils. In vitro and in vivo experiments [45] have demonstrated that peptidoglycan plays a crucial role in activating the systemic innate immune system, with NOD1 acting as a homeostatic regulator in this process. This NOD1-induced neutrophil activation can effectively eliminate *Streptococcus pneumoniae* or *S. aureus* from the respiratory tract [45]. In mice vaccinated with a live attenuated *Pseudomonas aeruginosa* vaccine, IL-17 was detected in bronchoalveolar lavage fluid (BALF) 6 h after infection with a heterologous LPS-bearing *P. aeruginosa* strain, which was associated with more rapid neutrophil recruitment to the lungs [46]. Although inoculation with an unrelated biological *Escherichia coli* also increased neutrophil numbers, this was not enough to reduce the bacterial load, indicating the non-redundant effect of antigen-specific Th17 cells [46]. These observations suggest that in respiratory diseases associated with microbial dysbiosis, simply increasing neutrophil numbers may not suffice to control infection, and that pathogen-specific immunomodulation merits further investigation in future studies.

#### 2.1.4. Innate Lymphoid Cells (ILCs)

ILCs are lymphocytes of the innate immune system that do not express specific antigen receptors (such as T-cell receptor or B-cell receptor) but can quickly respond to tissue injury and infection, and they play a key role in mucosal immunity, tissue homeostasis and inflammation regulation [47]. At present, some scholars divide ILCs into five subgroups according to their development and function, namely NK cells, ILC1, ILC2, ILC3 and LTi cells [48]. In mice infected with *Helicobacter pylori* and stimulated by IL-23, ILCs accumulate in the intestine and respond by secreting IL-22, IL-17 and IFN-γ [49]. ILC1 and NK cells that produce IFN-γ can reduce the risk of *Citrobacter rodentium* infection. Mice with depleted NK cells have a higher bacterial load, accompanied by more severe inflammation, and lower levels of IFN-γ, TNF-α, IL-12 and pathogen-specific IgG in the colon [50]. ILC3 in the intestine is the main source of IL-22. In the mouse model, IL-22 can enhance the tight junction of the intestinal epithelial barrier, induce the secretion of antimicrobial peptides and mucus and induce fucosylation of intestinal epithelial cells to maintain the ecological stability of intestinal microbiota [49]. IL-22 plays a role in protecting the respiratory tract from bacterial infections, and this protective effect is ILC3-dependent [51]; IL-22 can inhibit apoptosis in bronchial epithelial cells and reduce damage to the pulmonary barrier [52]. Therefore, we hypothesize that ILC3 may contribute to the homeostasis of the respiratory microbiota via IL-22.

### 2.2. Adaptive Immunity

The disorder of respiratory tract microbiota will not only directly activate the pro-inflammatory response of the innate immune system but also shift the immune response from rapid, non-specific innate immunity to highly specific adaptive immunity via antigen presentation. This key transition process mainly depends on the adaptive immune response mediated by B cells and T cells. B cells recognize specific antigens through B-cell receptors (BCRs) expressed on their surface, while T cells interact with MHC–antigen complexes on the surface of antigen-presenting cells (such as DCs) through a T-cell receptor (TCR) [32]. Against the background of respiratory tract microbiota imbalance, continuous antigen stimulation may contribute to polarization imbalance of Th1/Th2/Th17 cell subsets or induce excessive activation of B cells to produce autoantibodies, thus participating in the occurrence and development of chronic respiratory inflammation, allergic diseases and even autoimmune lung diseases.

#### 2.2.1. B Cell

B cells (B lymphocytes) are a key component of the adaptive immune system. They are mainly responsible for humoral immunity, derive from hematopoietic stem cells in bone marrow, and are broadly divided into plasma cells and memory B cells. In clinical studies, it was found that [53] lower serum IgG levels are significantly associated with reduced bacterial diversity in the lower respiratory tract, suggesting that low IgG may serve as a potential biomarker or mechanistic factor involved in the process of lower respiratory tract dysbiosis in patients with COPD. In patients with CF, activated CD69^+^B cells, CD4^+^CD8^+^T-cell subtypes and regulatory B cells decrease with the increase in pathogens (such as *Staphylococcus* and *Mycobacterium*); the concentration and fraction of PD-L1^+^B cells, granulocytes, monocytes, CD69^+^neutrophils and Tc17 cells are positively correlated with the increase in pathogen abundance [31]. These observations indicate that B cells and their subsets play complex and multifaceted roles in respiratory tract microbiota–immune crosstalk through both humoral immunity and immunoregulatory functions.

#### 2.2.2. T Cell

T cells (T lymphocytes) are key cells in the immune system and the core components of adaptive immunity. They originate from hematopoietic stem cells in the bone marrow and then migrate to the thymus to mature. The main functions of T cells include recognizing and clearing infected cells, regulating the immune response and forming immunological memory. Once T cells are activated, they expand and are recruited to the infected lung, where they are thought to mediate the control of bacterial growth through IFN-γ-induced activation of infected phagocytes. Allergic asthma is mainly caused by an excessive Th2 immune response and is also known as an IgE-mediated chronic inflammatory disease of the respiratory tract [54]. The lymph gland of CF patients contains CD4^+^IL-17^+^IL-22^+^T cells specific for *P. aeruginosa* antigen [55]. The T-cell response is dysregulated in CF patients, and it changes to a Th2- and Th17-dominated immune response [56]. In patients with CF and chronic infection, this effect depends on both quantitative and qualitative impairments in Treg cell homeostasis following *P. aeruginosa* infection [57]. In the primate animal model, CD4^+^T cells were identified as a key component of the *Mtb*-containing immune response during the incubation period [14]. Studies in clinical settings, in mouse and rabbit models, have shown that CD8^+^T cells account for 40% of the cells in *Mtb* human pulmonary granuloma and can also play a role in controlling infection through TCR selection, clonal expansion and cell-mediated cytolysis [58,59]. An experimental study conducted on mice has shown that targeting the IL-17-CXCL13 pathway rather than the IFN-γpathway holds potential as a new strategy for improving mucosal vaccines against *Mtb*; when administered via the mucosal route, this vaccine preferentially induces Th17 cells and provides Th1-independent immune protection, although this research is still in the preclinical stage. IL-17 mediates the induction of C-X-C motif chemokine ligand 13 (CXCL13) in the lung, which is used to strategically locate CXCR5^+^(C-X-C motif chemokine receptor 5 positive) T cells produced by pro-inflammatory chemokines in the lymphatic structure, thus promoting early and effective macrophage activation and *Mtb* control [60]. The upregulated differentially expressed genes (DEG) CXCL13, CR2, CD19, CCL19, CD79A and TLR10 were involved in the immune response of *Mtb*. A protein–protein interaction network has been established, indicating that CXCL13 is more likely to interact with CCL19, as well as CD79A and CD19, CD19 and CR2 [61]. These findings indicate that T-cell subsets exhibit disease-specific dynamic shifts in respiratory infections and chronic inflammatory conditions, and their functional status is closely linked to the composition of the respiratory microbiota and pathogen burden.

**Figure 2:**
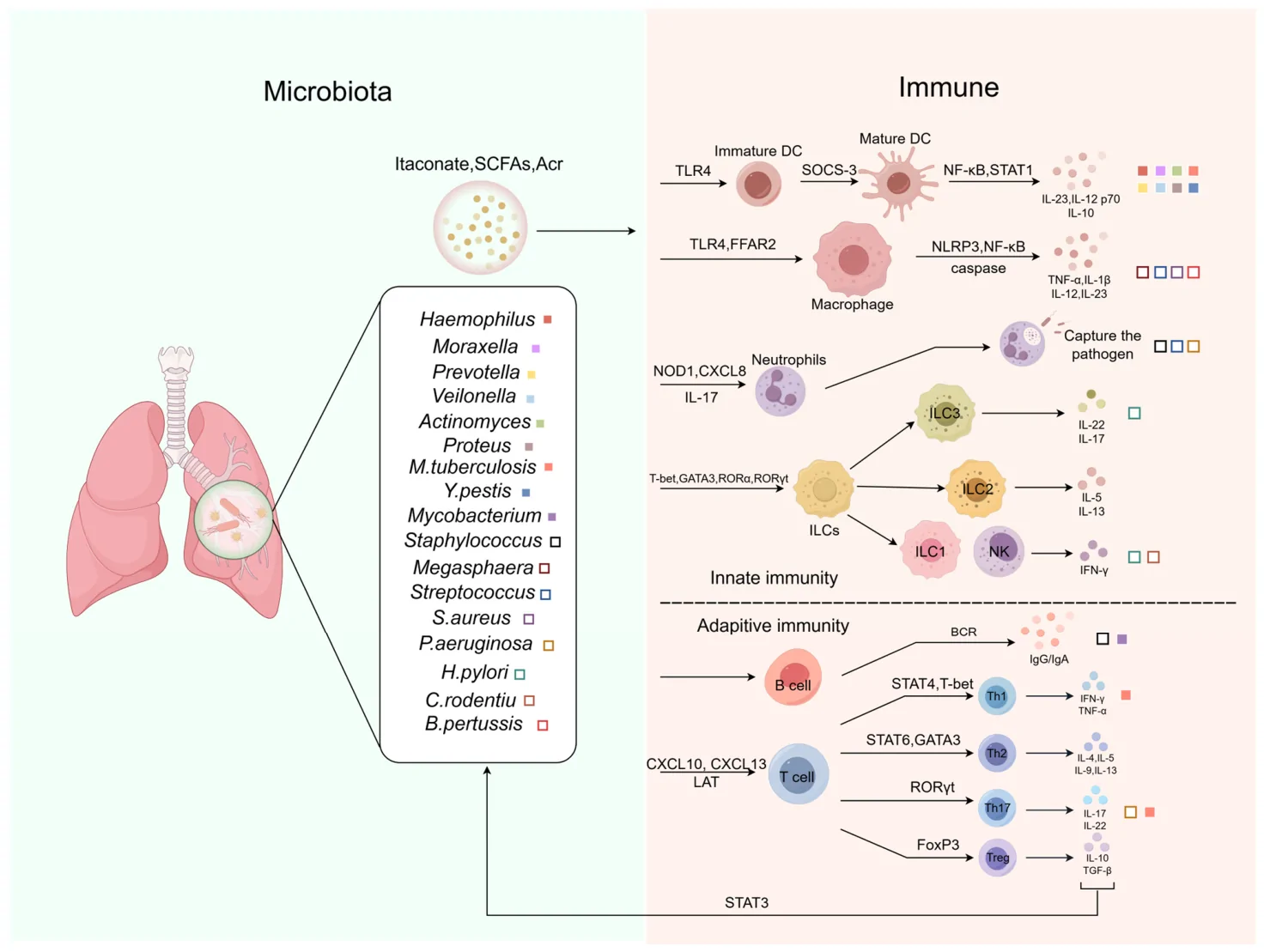
Interaction between respiratory tract microbiota and immune response. The square symbol indicates an increase in immune cells and cytokine secretion, corresponding to a microbial shift or vice versa. (The dotted line is used to distinguish between innate immunity and adaptive immunity.) The figure was created with Figdraw.com. https://www.figdraw.com/#/paint_index_v2. (accessed on 17 August 2026).

Figure 2 summarizes the bidirectional interaction network between respiratory tract microbiota and DCs, macrophages, neutrophils, ILCs, B cells, and T cells. During infection or dysbiosis, the network reconstructs towards a pro-inflammatory direction to clear pathogens, forming a vicious cycle of inflammation, immune suppression, and dysbiosis. There are significant differences in the stimulating ability of different bacterial genera on immune cells, indicating that the composition of microbial communities not only reflects the infection status but also actively participates in the shaping of the immune microenvironment and disease progression. Collectively, this framework suggests that microbiota–immune status assessment might serve as a basis for guiding future research, though its clinical utility remains unclear.

## 3. Dysbiosis-Related Respiratory Diseases

When the dynamic balance of the microbiota–immune axis is disrupted by internal or external factors, the composition, function, or metabolic output of the microbiota may change. In recent years, a wealth of evidence has established a close link between dysbiosis and the pathophysiological processes of various respiratory diseases. This section focuses on representative respiratory diseases, such as COPD and asthma, and aims to analyze the characteristic patterns of microbiota dysbiosis in each disease and to explore the implications of this microbiota–immunity–disease axis for developing treatment strategies.

### 3.1. Chronic Obstructive Pulmonary Disease (COPD)

COPD is a global health epidemic and the fourth leading cause of death worldwide. It is characterized by an irreversible decline in lung function due to airway inflammation, emphysema and alveolar destruction. COPD is heterogeneous, presenting with distinct clinical phenotypes that are underpinned by different pathophysiological mechanisms, termed endotypes [62]. The dominant bacteria in the lungs of patients with mild and moderate COPD are *Actinomycetes*, the dominant bacteria in the lungs of patients with severe COPD are *H. influenzae*, and the dominant bacteria in sputum are *Proteus*, while the proportion of *Actinomycetes*, *Clostridium* and *Bacteroides* has decreased [63,64]. Patients with high abundance of *Haemophilus* and *Streptococcus* in sputum samples have higher mortality risk [65]. Larsen et al. [29] divided the bacteria into three groups according to the immune stimulation ability of the strains to DC cells through principal component analysis: high irritant bacteria (*Haemophilus* and *Moraxella*), medium irritant bacteria (*Prevotella* and *Veroniella*), and weak irritant bacteria (*Actinomyces*) in vitro. The most common isolated bacterial microorganisms in patients with acute exacerbations of COPD include “*H. influenzae* (11% of all acute exacerbation subjects), *S. pneumoniae* (10%), *M. catarrhalis* (10%) and *P. aeruginosa* (4%), and Gram-negative bacteria are less common” [11]. The main taxonomic groups reported in COPD patients’ lungs include *Pseudomonas*, *Veroniella*, *Prevotella*, *Fusobacterium*, *Haemophilus* and *Streptococcus* [66]. Studies have found that patients with acute exacerbations of COPD carry high levels of the well-established pathogenic bacteria, including *P. aeruginosa, H. influenzae,* and *K. pneumoniae* [67]. In in vitro experiments, *H. influenza* infection induces the expression and activation of NLRP3 in human lung tissue, which may be the mechanism of COPD deterioration triggered by infection [68]. The respiratory tract microbiota of COPD patients is mainly composed of pathogenic bacteria such as *Haemophilus*, *Streptococcus*, and *Pseudomonas*, and their abundance is closely related to the risk of acute exacerbation and mortality.

### 3.2. Asthma

Asthma is a chronic inflammatory disease of the airway, characterized by cough, sneezing, wheezing, dyspnea, reversible airflow obstruction and other observable clinical features [69]. Endotypes correspond to the underlying molecular and immune mechanisms of phenotypes. For complex endotypes, they can be divided into type 2 or non-type 2 asthma [70]. According to the nature of asthma triggers, they can be roughly divided into allergic or non-allergic. *Acinetobacter*, *Sphingomonas*, *Pseudomonas* and *Lactobacillus* are pathogenic bacteria of allergic asthma, while *Akkermansia* and *Prevotella -6* are probiotics in the lung tissue of asthmatic mice [71]. In clinical studies of asthma patients, there are potential pathogenic bacteria in the upper and lower respiratory tract, such as *Haemophilus*, *Streptococcus*, *Moraxella*, *Klebsiella* and *Rickettsia*, which may be implicated in the prognosis of asthma and other respiratory diseases [72]. Clinical studies have shown that *S. pneumoniae*, *S. aureus*, *M. catarrhalis*, *P. aeruginosa* and *H. influenza* are associated with asthma exacerbation and development [73,74]; the respiratory microbiota of asthma is mainly composed of pathogenic bacteria such as *Haemophilus*, *Streptococcus*, and *Moraxella*; and their abundance is closely associated with the risk of exacerbation and disease progression. Although there are differences in the immunopathological mechanisms between COPD and asthma, specific bacterial genera may play a common role in promoting disease progression by driving airway inflammation and immune imbalance in both diseases.

### 3.3. Cystic Fibrosis (CF)

CF is a monogenic disease associated with a mutation in the *CFTR* gene and is one of the most common autosomal recessive diseases. *CFTR* encodes chloride channels that are highly expressed in epithelial cells of various tissues, making CF a multi-organ disease characterized by chronic airway infection, pancreatic dysfunction and abnormal sweat chloride. Several pathogens commonly associated with CF infection include *Pseudomonas*, *Stenotrophomonas*, *Mycobacterium*, *Haemophilus* and *Staphylococcus*, and fungi from *Aspergillus* and yeast. Among patients with CF, the relative abundance of *Mycobacterium*, *Staphylococcus* and *Veillonella* showed the most significant correlation with different immune cells, while *Pseudomonas* showed the lowest correlation [31]. The above research suggests that the impact of CF microbiota on host immunity is species-specific. This difference may stem from the varying abilities of different pathogens to activate host immune pathways.

### 3.4. Tuberculosis (TB)

TB is an ancient and serious infectious disease related to *Mtb*. It is characterized by a strong immune response and long-lasting interaction between host and pathogens. Although in recent decades, with the continuous expansion of vaccine research and development, new diagnostic tests and drugs, TB is still one of the main causes of death caused by a single pathogen before the COVID-19 pandemic [75]. *Mtb* accounts for the highest proportion in the lung tissue of TB patients, followed by *Megasphaera stantonii*, *Vibrio vulnificus* and *Furfurilactobacillus rossiae,* whereas *S. aureus* is dominant in the lung tissue of non-TB patients [61]. While the microbiota–immune axis in TB appears to exhibit features distinct from those seen in other chronic respiratory diseases, whether this difference warrants a departure from broad-spectrum anti-infective strategies toward *Mtb*-targeted immune reconstitution remains an open question that requires further investigation.

### 3.5. Pneumonia and Bronchiectasis

Pneumonia refers to the pulmonary inflammation caused by pathogen infection, physical and chemical factors or immune injury, which mainly involves the alveoli, terminal airway and pulmonary interstitium, and is characterized by fever, cough, dyspnea and pulmonary infiltrating shadow. It can be divided into bacterial pneumonia, viral pneumonia, atypical pneumonia, fungal pneumonia and aspiration pneumonia, as well as radiation pneumonia and chemical pneumonia due to special reasons. The main pathogenic bacteria of pneumonia are *S. pneumoniae* and *H. influenza type B* [76]. Mice infected with *S. pneumoniae* after 6 months of exposure to cigarette smoke had reduced pulmonary microbial diversity but increased levels of *Lactobacilliaceae* in the upper respiratory tract [77].

Bronchiectasis is a chronic airway disease that is characterized by abnormal and permanent expansion of bronchi and bronchioles, leading to repeated infection, chronic cough and copious pus and sputum [78]. The enrichment of *Neisseria* in the respiratory tract microbiota is significantly associated with more frequent acute exacerbations in patients, and it was shown that *Neisseria subflava* induced inflammation of primary epithelial cells and mouse lungs and destroyed epithelial integrity. Collectively, pneumonia and bronchiectasis share a common microbiota–immune interaction pattern characterized by opportunistic pathogen enrichment.

COPD, asthma, and CF exhibit partially overlapping patterns of dysbiosis, all characterized by an enrichment of opportunistic pathogens (such as *Haemophilus*, *Streptococcus*, *M. catarrhalis*, and *P. aeruginosa*) and a reduction in the abundance of commensal bacteria such as *Prevotella* and *Veillonella*, suggesting the existence of a potential core dysbiosis pattern that may contribute to the inflammatory processes underlying multiple respiratory diseases (Table 1). However, the term of core dysbiosis pattern used here is a conceptual framework to facilitate microbiota–disease crosstalk but not intended to imply a universally reproducible microbial signature, and the validity of this pattern requires further clarification. The TLR4 and CXCL8 signaling pathways are activated in most of these diseases, suggesting that excessive activation of the innate immune system is a central component of the respiratory tract microbiota–immune interaction, whereas abnormalities in the TCR/BCR signaling pathways in CF and TB reflect the involvement of the adaptive immune system. The integration of microbiota and immune network profiling may inform future exploration in related diseases, but its clinical and translational benefit remains hypothetical at this stage.

## 4. Microbiota-Based Therapeutic Strategies for Respiratory Diseases

The role of microbiota dysbiosis in driving the onset and progression of respiratory disease has been widely recognized. Restoring a healthy microbial ecosystem may therefore represent a viable therapeutic strategy. In this section, we review the current evidence on intervention in regulating the respiratory tract microbiota (Table 2) and analyze the possibility of microbiota–immune interaction targets for clinical translation.

### 4.1. Herbal Medicines

In the mouse model, Radix isatidis aqueous extract can inhibit the TLR3 pathway and neuraminidase activity, thereby protecting mice and host cells from acute lung injury and apoptosis induced by influenza virus or LPS, and exhibits significant bactericidal activity against *S. aureus*, *E. coli*, *Bacillus subtilis*, and *Salmonella* [79,80,81]. The Daqing Formula can significantly reduce the aggravation of allergic asthma and dysbiosis caused by OVA in mice, and its mechanism may be related to restoring the balance of Th1/Th2. By inhibiting the pathway mediated by GSK-3β, it can inhibit the proliferation and migration of EOS sensitized by OVA+LPS, induce apoptosis, and reduce the release of a variety of inflammatory cytokines [71]. In the rat model, Gan-du-qing alleviates PM2.5-induced pulmonary dysbiosis; *Lactobacillus* was higher than in the PM2.5 group after GDQ intervention, while *Pseudomonas* and *Mycoplasma* were lower than in the PM2.5 group [82]. The Yifei Sanjie Formula alleviates pulmonary inflammation in COPD rats through the NLRP3/caspase-1/IL-1β signaling pathway, reduces the abundance of *Ralstonia* and *Mycoplasma*, and upregulates the abundance of *Halomonas*, *Dietzia*, and *Nesterenkonia* [83]. Yangyin Qingfei Oral Liquid regulates the upper respiratory tract microbiota in patients with laryngeal cough, significantly reducing the abundance of microorganisms such as *Streptococcus* and *Haemophilus*, while increasing the abundance of *Veillonella* [84]. Herbal medicines and their compounds are characterized by multiple components, multiple targets, and a networked structure and have the potential to systematically modulate the microbiota–immune axis, but this concept is still in the preclinical stage and requires further validation.

### 4.2. Probiotics

Liu et al. proved that oral probiotic *Pediococcus pentosaceus* SMM914 can delay the progress of COPD by reducing lung oxidative stress. Strain SMM914 increases the abundance of SCFA-producing probiotics and activates antioxidant metabolism. At the same time, strain SMM914 synthesized L-tryptophan amide, 5-hydroxy-L-tryptophan and 3-sulfurous-L-alanine, thereby enhancing the tryptophan melatonin pathway and increasing 6-hydroxymelatonin and taurine in the lung environment. This regulation amplified the secretion of endogenous anti-inflammatory factors, reduced the polarization of macrophages to the M1 phenotype, and ultimately reduced the oxidative stress of COPD mice [85]. *Rothia mucilaginosa*, a bacterium primarily colonizing the oral cavity, has been detected in the lower respiratory tract of patients with chronic respiratory diseases; this bacterium can suppress pro-inflammatory responses induced by pathogens or LPS, which has been validated from multiple perspectives, including clinical, in vitro, and animal studies [86]. Although specific strains have demonstrated the potential to modulate the immune system through metabolites in animal models, current research is largely limited to the preclinical stage and lacks validation in large-scale clinical cohorts.

### 4.3. Other Treatment Methods

Clinical studies have found that [87] hereditary mannose-binding lectin (MBL) deficiency can prevent *Haemophilus* infection, increase the diversity of lung microbiota, and modulate the composition of lung ecology. COPD patients without MBL can reduce the risk of acute exacerbation. Increasing consumption of green vegetables and fruits rich in SCFAs is related to the reduction of the risk of COPD and the improvement of symptoms such as dyspnea in patients with COPD [88]. Detected in patients with CF, high concentrations of SCFAs inhibit the growth of *P. aeruginosa*, and the inhibitory effect is enhanced at lower pH, while low concentrations of SCFAs can promote the growth of bacteria in vitro [89]. Antibiotic treatment of COPD patients improved the diversity of respiratory tract microbiota, reduced the relative abundance of *Proteobacteria*, and increased the relative abundance of *Firmicutes*. These changes have restored the ecological imbalance related to COPD in some way, and its effect can still be maintained after the cessation of antibacterial treatment [90]. Among patients with COPD, the effect of corticosteroids is different from that of antibiotics and is related to the reduction of species diversity and the increase in *Proteobacteria* relative to *Firmicutes*. This corresponds to the increase in *Haemophilus* and *Moraxella* and the decrease in *Streptococcus*, which are related to the pathogenesis of COPD [91]. Inoculation of live attenuated *P. aeruginosa* vaccine can protect mice from the attack of LPS heterologous strains of *P. aeruginosa* [46].

Potential targets involved in modulating the commensal microbiota and immune system for future interventions in respiratory diseases have been summarized; however, these targets have yet to be validated in clinical trials. *Prevotella* can partly reduce the production of inflammatory factors by DCs induced by *Haemophilus* in vitro [29]. This suggests a potential immunoregulatory mechanism; *Prevotella* can be considered as a means or indicator for the treatment of respiratory diseases (COPD, asthma, etc.) associated with *Haemophilus*. In the mouse model, The Acr protein produced by *Mtb* plays a certain role in the immune network [12]. Since *Mtb* is the predominant bacterium in patients with CF and TB, future clinical interventions may be achieved by targeting downstream signaling molecules such as SOCS-3 or by developing vaccines that target the Acr protein. Animal models have demonstrated that NOD1-induced neutrophil activation can effectively clear *S. pneumoniae* or *S. aureus* from the respiratory tract; these two respiratory commensal bacteria are associated with many respiratory diseases [46]. Meanwhile, peptidoglycan plays a central role in immune regulation through its receptor network (including NOD1 and NOD2), which can both enhance immune defenses and suppress pathological inflammation, making it a therapeutic target well worth further investigation. As mentioned earlier, IL-22 plays a role in repairing the mucosal barrier and combating infections. However, it is worth noting that a study in which mice were administered recombinant IL-22 disrupted the homeostasis of ILCs in the lungs [52], suggesting that IL-22 plays a more complex role in respiratory immunity, which warrants further investigation. However, none of the above studies have reached the clinical application stage, and extensive further experimentation will be needed to explore their feasibility.

**Table 2:** Strategies for treating diseases through interventions of respiratory microbiota.

Intervention	Samples Source/Model	Regulation of Respiratory Tract Bacteria	Immunocyte	Molecular Targets (Proteins, Factors)	References
Radix isatidis aqueous extract	LPS induced acute lung injury in mice	*S. aureus*, *E. coli*, *B. subtilis*, *Salmonella* ↓	macrophages, neutrophils	IL-6, IL-1β, iNOS, COX-2, TNF-α, caspase-11, PGE2 ↓	[79,80,81]
Chinese herbal compound Daqing formula	allergic asthma model in mice	*Moraxella*, *Streptococcus*, *Ackermann*, *Prevotella*, *S. aureus*, *K. pneumoniae*, *S. pneumoniae* ↓	macrophages, eosinophils, Th1 ↑, Th2, CD4^+^	IFN-γ, IL-12 ↑; IL-4, IL-5, IL-25, IL-33, p-GSK-3β, p-p65, nuclear β-catenin, p-STAT3 ↓	[71]
Gan-du-qing	rat model of PM2.5 exposure	*Pseudomonas*, *Mycoplasma* ↓, *Lactobacillus* ↑	Th17/Treg	phospholipase D signaling, cytochrome P450-mediated metabolism of xenobiotics, glutathione metabolism	[82]
Yifei Sanjie Formula	rat model of COPD	*Ralstonia, Mycoplasma* ↓, *Halomonas*, *Dietzia*, *Nesterenkonia* ↑	neutrophil	NLRP3/caspase-1/IL-1β signaling pathway	[83]
Yangyin Qingfei Oral Liquid	laryngeal cough patients	*Streptococcus, Haemophilus* ↓, *Veillonella* ↑	-	-	[84]
Oral probiotics *P. pentosaceus* SMM914	mice with COPD	probiotics producing SCFAs and antioxidant metabolism ↑	M1-type macrophages ↓	TNF-α ↓, IFN-γ ↓, IL-10 ↑	[85]
*R. mucilaginosa*	LPS induced inflammation in mice	*P. aeruginosa* ↓	macrophages, granulocytes	IL-6, IL-8, (MIP)-2, IL-1β ↓	[86]
MBL genetic defects	COPD patients	*Haemophilus* ↓	macrophages	IL-1β, TNF-α ↓	[87]
SCFAs	In vitro	*P. aeruginosa* ↓	macrophages, granulocytes	inducible nitric oxide synthase ↓	[89]
Prophylactic use of antibiotics	COPD patients	*Proteobacteria, Firmicutes*	-	-	[90]
Use corticosteroids	COPD patients	*Haemophilus*, *Moraxella* ↑, *Streptococcus* ↓	eosinophils ↑	cysteinyl leukotrienes ↑	[91]
Live attenuated *P. aeruginosa* vaccine	acute fatal pneumonia model in mice	*P. aeruginosa* (*PAO1*) ↓	neutrophils, Th17	IL-17 ↑	[46]

Table footer: the up and down arrows in the table represent changes in bacterial abundance and molecular levels. LPS: lipopolysaccharide; COPD: chronic obstructive pulmonary disease; MBL: mannose-binding lectin; SCFAs: short-chain fatty acids.

## 5. Future Directions and Recommendations

The investigation of the relationship between respiratory immune cells and bacterial microbiota helps to provide strategies for disease intervention from “antibacterial” to “homeostasis regulation”, that is, indirect regulation by interfering with the microbiota and immunity rather than simple use of broad-spectrum antibiotics for indiscriminate killing. Growing evidence indicates that clinicians are paying attention to the respiratory tract microbiota, using nasal swabs [22], throat swabs [84], sputum samples [65], and BALF [24] to detect changes in the respiratory tract microbiota. In a double-blind clinical trial conducted in Vietnam [92], the use of nasal-spray *Bacillus* probiotics as a treatment for children with pneumonia successfully shortened the duration of pneumonia symptoms and reduced the load of *S. pneumoniae* and *H. influenzae* in the respiratory tract, but there are currently few clinical cases involving the direct regulation of the respiratory tract microbiota.

In view of the crosstalk of many microorganisms between the intestine and the lungs, the intestinal microbiota can be modulated by oral probiotics, prebiotics or microbiota transplantation, which can further shape the lung microbiota and immune homeostasis [93]. Current double-blind clinical trials have shown that a combination of lactic acid bacteria can improve respiratory symptoms in COPD patients and reduce inflammation [65]. Clinical studies have shown that three consecutive months of probiotic treatment can help maintain the homeostasis of the gut microbiota and the immune system, thereby reducing the frequency of recurrent respiratory infections in children [94]. The above evidence indicates that the metabolites of microbiota, such as SCFAs, are promising targets in the treatment of respiratory disease. Direct pulmonary administration still faces technical challenges, and gut-targeted interventions via the gut–lung axis for modulating respiratory tract immunity have not yet been validated. Further research is needed to determine whether this approach can produce clinical effects.

The strategy of combining multi-omics integration and functional verification may help systematically identify key targets in the network of respiratory tract microbiota and immune system. Through cross-sectional correlation analysis combining metagenomics, metabolomics, and single-cell sequencing, key bacteria and their associated immune cell populations can be preliminarily identified. Current clinical studies are using next-generation sequencing (NGS) technology to sequence the gut microbiota of patients with bronchiectasis and compare the results with inflammatory markers to examine the interaction between the respiratory tract microbiota and the immune system in these patients [95]; this is combined with metagenomics and metabonomics analysis to characterize the changes in intestinal microbiota composition and metabolism in mice after respiratory tract infection [96].

Current methods for testing respiratory tract microbiota have limitations, such as differences in sampling sites of the upper or lower respiratory tract, and methodological approaches for sequencing or bioinformatics pipelines across studies may influence the observed microbial composition. Additionally, throat swabs could not reflect the microbiota of the lower respiratory tract [97], sputum samples are heavily contaminated with host DNA [98], and although BALF is the gold standard for testing lower respiratory tract microbiota [99], it is an invasive procedure with low patient acceptance and requires a high level of skill from the operator. If sputum samples are used, the challenge of removing host DNA must be addressed [97]; alternatively, tracheal aspiration could be used in place of BALF [100], but this approach remains at the theoretical stage and has not been clinically validated. After further exploration in the future, machine learning techniques might help to integrate respiratory microbiota with host immune responses to facilitate the diagnosis of respiratory infections [101]; this technology is not yet mature and still needs to be validated.

Most existing studies on the relationship between the microbiota and the immune system are limited to correlational analyses, making it difficult to determine whether the microbiota affects the immune system and leads to disease, or whether immune system dysfunction causes microbiota dysbiosis. While these studies are essential for hypothesis generation, their findings cannot directly translate to clinical research. There is a technical bottleneck in establishing an animal model of humanized microbiota–immune interaction, as well as the respiratory tract microbiota and organoid co-culture systems [102]. High-throughput functional screening could be conducted using CRISPR-based screening or microbial metabolite libraries [103], but the application of such methods in the field of the respiratory tract microbiota is still in its infancy, and much work remains to be done before they can be translated into clinical practice.

In conclusion, we identified several pathogenic bacteria (such as *Haemophilus*, *Streptococcus, Moraxella*, and *Staphylococcus*) associated with respiratory diseases; the respiratory commensal microbiota (such as *Prevotella* and *Veillonella*) may contribute to maintaining immune homeostasis or regulate disease progression, but this requires further investigation. We also indicated immune pathways—primarily mediated by TLRs, NOD-like receptors, or the NLRP3 and STAT signaling pathways—that link the respiratory tract microbiota to the host immune system and are implicated in various diseases. However, we emphasize that most proposed interventions remain preclinical, and their clinical translation requires further validation. Based on this “microbiota–immunity–disease” link, the findings may provide a foundation for developing novel therapies for chronic respiratory diseases, such as COPD, asthma, and pneumonia, as well as recurrent and severe respiratory infections.

## Figures and Tables

**Table 1:** The respiratory tract microbiota affects host immune system and diseases.

Diseases	Respiratory Bacteria Status	Affected Immune Cells	Evidence Type	Molecular Changes (Proteins, Factors)	Key Signaling Pathway	References
COPD	*Actinobacteria* ↓	DCs	in vitro	IL-23, IL-12p70 ↓, IL-10 ↑	TLR4	[29]
	*Haemophilus* ↑	DCs	in vitro	IL-23, IL-12p70, IL-10 ↑	TLR4	[29]
		macrophages	in vitro	IL-1β, IL-18	NLRP3	[68]
		neutrophils	patients	IL-8 ↑	CXCL8	[65]
	*Streptococcus* ↑	neutrophils	patients	IL-8 ↑	CXCL8	[65]
	*M. catarrhalis* ↑	DCs	in vitro	IL-23, IL-12p70, IL-10,	TLR4	[29]
		neutrophils	patients	IL-8 ↑	CXCL8	[65]
	*Prevotella* ↓	DCs	in vitro	IL-23, IL-12 p70, IL-10 ↑	TLR4	[29]
	*Veillonella* ↓	DCs	in vitro	IL-23, IL-12p70, IL-10 ↑	TLR4	[29]
Asthma	*Haemophilus* ↑	DCs	in vitro	IL-23, IL-12p70, IL-10 ↑	TLR4	[29]
		macrophages	in vitro	IL-1β, IL-18 ↑	NLRP3	[68]
	*Streptococcus* ↑	neutrophils	patients	IL-8 ↑	CXCL8	[65,72]
	*P. aeruginosa* ↑	neutrophils, T cell	animal model	IL-17 ↑	-	[46]
	*Prevotella* ↓	DCs	in vitro	IL-23, IL-12p70, IL-10 ↑	TLR4	[29]
	*S. aureus* ↑	macrophages,	animal model	CCR2 ↑	TLR2	[37]
		neutrophils	animal model	IL-6 ↑	NOD2	[39]
CF	*Haemophilus* ↑	DCs	in vitro	IL-12p70 ↑	TLR4	[29]
	*P. aeruginosa* ↑	CD4^+^ IL-17^+^ IL-22^+^ T cell	patients	IL-17, IL-22 ↑	-	[55]
		neutrophils	patients	IL-17 ↑	-	[56]
	*Mycobacterium* ↑	DCs, Th17	in vitro	IL-17 ↑	Acr protein	[12]
	*Veillonella* ↑	DCs	in vitro	IL-23, IL-12p70, IL-10 ↑	TLR4	[29]
TB	*Mycobacterium* ↑	DCs, Th17	in vitro	IL-17 ↑	Acr protein	[12]
		B cell	patients	IgG, IgA ↓	BCR, NF-κB	[61]
Pneumonia	*Streptococcus* ↑	macrophages, neutrophils	animal model	IL-6 ↑	CXCL8, TLR4	[77]
	*Haemophilus* ↑	DCs	in vitro	IL-23, IL-12p70, IL-10 ↑	TLR4	[29]

Table footer: the up and down arrows in the table represent changes in bacterial abundance and molecular levels. COPD: chronic obstructive pulmonary disease; CF: cystic fibrosis; TB: tuberculosis; DC: dendritic cell.

## Data Availability

No new data were created or analyzed in this study. Data sharing is not applicable to this article.
